# Level of personality functioning among outpatients with predominant anxiety symptoms

**DOI:** 10.1192/j.eurpsy.2024.1361

**Published:** 2024-08-27

**Authors:** A. Bogdanovska Toskic, S. Arsova Hadzi-Angjelkovska

**Affiliations:** ^1^Psychiatry, General Hospital Kumanovo, Kumanovo; ^2^Psychiatry, University Clinic of Psychiatry, Skopje, North Macedonia

## Abstract

**Introduction:**

Dimensional diagnosis of personality disorders has as its main criterion the assessment of the level of functionality. And in patients with other diagnostic categories, there is a difference in the degree of functioning, as well as a difference in the course and prognosis of the disorder. The reason for such a different course may be the existence of a certain degree of personality dysfunctionality.

**Objectives:**

The aim of the study is to determine the prevalence of personality disorder in patients with neurotic disorder and predominantly anxiety symptomatology.

**Methods:**

A descriptive cross-sectional study was made to determine personality disorder in patients with neurotic disorder (F40-F48, excluding those where the disorder is related to stress F43) and predominantly anxiety symptomatology. The HAM-A scale was used to assess anxiety, and the LPFS-BF-2.0 was used to assess the level of personality functioning. The results were processed by descriptive statistical analysis.

**Results:**

The study included 25 individuals (*N* 25, 64% women), aged between 18 and 65 years (mean age 44.16, SD 13.20) with a diagnosed neurotic disorder. All subjects had elevated anxiety symptomatology, mean HAM-A score was 35.36 (SD 7.76). The assessment of the level of personality functioning with the LPFS-BF-2.0 gave the following results: 20% of people have a personality difficulty, 12% have a mild personality disorder, 32% have a moderate and 4% have a severe personality disorder.

**Conclusions:**

According to the obtained results, 68% of people with a neurotic disorder and a high degree of anxiety have a certain degree of personality dysfunction. The prevalence of personality disorder in individuals with neurotic disorder is high (48%). These results lead to the conclusion that people with pronounced anxiety often have a disruption in personality. In people with a high level of anxiety, an assessment should be made for the level of functioning of the person, as well as for the existence of a personality disorder, and the treatment should be adjusted according to the results obtained. In addition to the treatment of the emerging symptoms, the personality dysfunctions should also be treated.

**Disclosure of Interest:**

None Declared

